# Gastrointestinal Transit Time, Glucose Homeostasis and Metabolic Health: Modulation by Dietary Fibers

**DOI:** 10.3390/nu10030275

**Published:** 2018-02-28

**Authors:** Mattea Müller, Emanuel E. Canfora, Ellen E. Blaak

**Affiliations:** Department of Human Biology, NUTRIM School for Nutrition and Translational Research in Metabolism, Maastricht University Medical Centre, Universiteitssingel 50, 6229 ER, P.O. Box 616, 6200 MD Maastricht, The Netherlands; m.muller@maastrichtuniversity.nl (M.M.); emanuel.canfora@maastrichtuniversity.nl (E.E.C.)

**Keywords:** dietary fiber, gastrointestinal transit, microbiota, obesity, type 2 diabetes

## Abstract

Gastrointestinal transit time may be an important determinant of glucose homeostasis and metabolic health through effects on nutrient absorption and microbial composition, among other mechanisms. Modulation of gastrointestinal transit may be one of the mechanisms underlying the beneficial health effects of dietary fibers. These effects include improved glucose homeostasis and a reduced risk of developing metabolic diseases such as obesity and type 2 diabetes mellitus. In this review, we first discuss the regulation of gastric emptying rate, small intestinal transit and colonic transit as well as their relation to glucose homeostasis and metabolic health. Subsequently, we briefly address the reported health effects of different dietary fibers and discuss to what extent the fiber-induced health benefits may be mediated through modulation of gastrointestinal transit.

## 1. Introduction

Food intake activates several gastrointestinal (GI) processes. The transit of food through the stomach, small intestine and colon is crucial for digestion and absorption of nutrients. Upper intestinal transit includes gastric emptying (GE) and small intestinal motility and plays a major role in satiety and appetite regulation, glycemic control and gut hormone signaling [1,2]. Furthermore, the rate of lower intestinal i.e., colonic transit has a major impact on the gut microbiota [3], which may be involved in many physiological functions in energy and substrate metabolism, metabolic cross-organ signaling and insulin sensitivity [4]. Thus, altered GI transit may play a role in the etiology of metabolic diseases such as obesity and type 2 diabetes mellitus (T2DM) [5]. Food-based approaches are under investigation among which indigestible dietary fibers are long acknowledged to be beneficial in the prevention of chronic metabolic diseases. Indeed, observational studies showed that intake of dietary fibers from various plant sources decrease the prevalence of obesity and T2DM [6,7]. Nevertheless, data from human intervention studies are less consistent and may depend, among other factors, on type and amount of dietary fibers, overall diet composition and the metabolic phenotype of the individual [8]. The underlying mechanisms of the beneficial effects of dietary fibers are not completely understood, but the GI transit might play a role in these mechanisms. Indeed, dietary fibers may affect the GI transit of nutrients via various mechanisms depending on their physical-chemical properties (i.e., viscosity, fermentability and water solubility) [9]. This review discusses how GI transit may relate to glucose homeostasis and metabolic health and to what extent the positive metabolic effect of dietary fiber intake may be mediated by modulation of GI transit. First, we provide an overview on methodologies to assess GI transit. Then, we discuss the (patho)physiological consequences of an altered GI transit on glucose homeostasis, gut hormonal secretion and gut microbiota composition. Secondly, we review available human evidence of insoluble, soluble viscous and non-viscous fiber on glucose homeostasis. Finally, we discuss to what extent these effects may be mediated GI transit.

## 2. Gastrointestinal Transit: Methodology and (Patho)physiology

### 2.1. Gastric Emptying and Its Regulation

After ingestion, food passes through the esophagus and reaches within seconds the stomach. Initially, food intake induces gastric accommodation in the proximal part of the stomach thereby increasing its capacity to store ingested foods. Contractile activity patterns mix gastric liquids and push solid food particles towards the distal antrum and pylorus. The antrum repetitively contracts and grinds food particles against the closed pylorus facilitating mechanical and chemical break down of solid foods. Consequently, the pylorus opens to gradually release chyme with a size of 1–2 mm at a rate of 4 kcal/min into the duodenum; a process termed gastric emptying (GE) [10]. GE rate is often reported as initial lag phase (T_lag_, i.e., the phase between ingestion and start of emptying) and GE half-time (T_1/2_, i.e., the time at which half of the meal is emptied). GE rate adapts to energy density, volume and digestibility of the meal and differs greatly between solid and liquid foods. Macronutrients generally slow down GE, an effect that is mainly dependent on the caloric content of the macronutrient rather than type of macronutrient [2,11]. Non-caloric liquids are rapidly released from the stomach into the duodenum in an exponential manner. Solid food empties in a biphasic manner described by T_lag_ which takes few minutes up to an hour followed by a linear emptying phase [10]. The gold standard to measure GE rate is imaging with radionuclide scintigraphy. It visualizes the retention of a stable isotope labeled meal in the stomach [12]. Due to the exposure to ionizing radiation, other validated non-invasive methods have been developed using paracetamol or non-radioactive ^13^C stable isotopes. After ingestion of a ^13^C-isotope or paracetamol labeled meal, rapid duodenal absorption enables detection of ^13^CO_2_ in the expired breath or paracetamol in the blood, respectively [13].

The regulatory mechanisms coordinating GE rate are complex and involve the central nervous system (CNS), enteric motor neurons and the gastric smooth muscle cells. Normal GE rate is stimulated by contractile activity of the stomach and small intestine, which is coordinated by the CNS, the vagus nerve and neurohumoral peptides [14]. During the fasting and postprandial state, numerous neurohumoral gut peptides are secreted from enteroendocrine cells in the gastric and intestinal mucosa (i.e., ghrelin, cholecystokinin (CKK), glucagon-like peptide-1 and 2 (GLP-1, GLP-2) and peptide YY (PYY)). Food intake inhibits ghrelin secretion and stimulates secretion of CCK, PYY and GLP-1, which in turn modulates gastric and intestinal motility by activating receptors on sensory, vagal and intrinsic afferent neurons. Thus, GLP-1, PYY and CCK delay GE rate and induce satiety via central signaling [15,16,17,18]. This negative feedback loop controls food transit through the upper gut to optimize digestion and nutrient absorption.

### 2.2. Gastric Emptying and Blood Glucose Homeostasis

GE rate determines the appearance rate of glucose in the duodenum and its subsequent absorption and systemic appearance [19]. In healthy individuals [20,21], as well as in individuals with obesity [22] and T2DM [23], GE rate explained around 30% of the variance in peak circulating glucose concentrations after an oral glucose load. Notably, a higher initial GE rate is directly related to the initial rise in postprandial glucose concentrations [2]. Over time, elevated postprandial blood glucose may play a role in the development of insulin resistance and T2DM [24]. In the overweight or obese state, GE rate has been reported to be faster, similar or delayed compared to lean individuals [25,26]. However, a recent, large-scale cross-sectional analysis reported faster GE rates measured via scintigraphy and reduced fasting plasma PYY concentrations in overweight and obese compared to lean adults [27]. Therefore, it is intriguing to speculate that accelerated GE in obesity may lead to rapid increase in blood glucose, thus imposing a constant challenge to postprandial glucose homeostasis. However, data on altered GE rates in overweight and obesity is still inconclusive, which is likely due to different methodologies, test meal composition and study population heterogeneity.

To conclude, controlled release of nutrients from the stomach affects blood glucose appearance and in turn is regulated by nutrient-induced release of neurohumoral gut peptides. There are indications that GE rate is altered in overweight or obese individuals which might contribute to postprandial hyperglycemia. Thus, modulation of GE rate might be a potential target to modulate postprandial glycemia, thereby reversing or preventing cardiometabolic risk.

### 2.3. Small Intestinal Transit and Its Regulation

Food transit through the small intestine depends on GE rate and involves simultaneous propulsion, mixing and segmentation of chyme along all intestinal segments. Regional contractions mix the luminal content with digestive secretions and increase the contact between chyme and mucosal surface to optimize nutrient absorption. When the stomach is empty, organized repetitive contractions, termed the migrating motor complex (MMC), spread from the stomach along the small intestine. These contractions facilitate efficient nutrient absorption and evidentially cleansing of undigested food particles to prevent small intestinal bacterial overgrowth [28]. Small intestinal flow patterns are measured with intestinal manometry or impedance catheters that capture intestinal pressure waves and the transit of a food bolus. In the clinic and research setting, less invasive, indirect breath tests are usually used to measure small intestinal transit time. Breath tests measure oro-cecal transit, which describes the time from food ingestion to arrival of chyme in the caecum. After ingestion of an indigestible, fermentable carbohydrate (i.e., lactulose or inulin), exhaled breath hydrogen is measured in intervals up to 5 h. Once entered into the cecum, colonic bacteria rapidly ferment the indigestible carbohydrates leading to increased hydrogen excretion detectable in exhaled breath [29,30]. However, oro-cecal transit measured via hydrogen exhalation showed a high inter-individual variability indicating that it might only detect significant differences in case of extreme delayed/accelerated transit [31]. Recently, the wireless SmartPill has been introduced which measures intestinal transit time based on luminal pH differences along intestinal segments [32].

### 2.4. Small Intestinal Transit and Glucose Homeostasis

Small intestinal motility patterns likely influence the extent of glucose absorption, but little is known about the underlying mechanisms. Luminal carbohydrate breakdown, small intestinal flow patterns and mesenteric blood flow may play a role in intestinal glucose absorption. The major part of glucose is absorbed in the proximal intestine via luminal sodium-glucose co-transporter (SGLT1) and glucose transporter 2 (GLUT2) at the baso-lateral membrane [33]. Drug-induced attenuation of small intestinal luminal flow markedly reduced the early postprandial plasma glucose peak in healthy humans [34]. Other studies using nutrient infusion into different sites of the small intestine indicated that increasing the intestinal area that is exposed to nutrients may have beneficial effects on postprandial glycemia, satiety, hunger and gut peptide responses [35].

Only a few studies have assessed small intestinal transit time in overweight and obese adults. In obese women and lean participants, intestinal contents were sampled via a tube inserted in the proximal 70 cm of the intestine. After intake of a high-fat liquid test meal, the energy content of the luminal sample was lower in obese women compared to lean participants, indicating a more effective rate of intestinal nutrient absorption [36]. Upon prostaglandin-induced stimulation, intestinal smooth muscle strips from obese showed an increased contractility measured with isometric transducers compared to intestinal muscle strips from lean controls [37]. In contrast, the oro-cecal transit of 100 mL water measured via lactulose breath test was slower in obese compared to normal weight controls [38]. Another study reported no differences in oro-cecal transit in obese compared to lean participants despite higher postprandial glucose concentrations after a liquid test meal in individuals with obesity [22]. In addition, no association was found between body mass index (BMI) and oro-cecal transit assessed by scintigraphy in a cross-sectional analysis including lean and obese individuals [39].

To summarize, evidence suggests a role of small intestinal transit in postprandial glucose absorption. However, data on altered small intestinal transit in obesity is yet inconclusive. Among other factors, studies lack standardized methodology such as harmonized test meal compositions. Further, well-designed human studies are warranted to study putative differences in small intestinal transit patterns in overweight/obese vs. normal weight individuals including their contribution to glycemic control.

### 2.5. Colonic Transit and Its Regulation

The main function of the colon is to absorb water and electrolytes, a process much slower than small intestinal nutrient absorption, and storage of food waste [40]. The enteric nervous system largely controls colonic motor function. Colonic motor activity occurs in high-amplitude propagated contractions compartmentalizing colonic segments which eventually results in stool formation [41]. Gut peptides involved in colonic motility are somatostatin, neurotensins, motilin and corticotrophin-releasing factor (CRF) [42]. There is no consensus on the golden standard for measuring colonic transit. In clinic and research settings, scintigraphy, wireless SmartPills and radio-opaque marker methods are commonly used. The latter involves ingestion of a defined number of radio-opaque markers in 24 h intervals. After 4 to 7 days of ingestion, an abdominal X-ray is taken and colonic transit time is calculated based on the amount of markers visible on the X-ray [43]. Stool consistency assessed via the Bristol Stool Chart (BSC) is often used as a proxy for colonic transit. Individuals classify stool consistency from very firm to loose stool. Low BSC scores, i.e., firm stool, indicate slow colonic transit and high BSC scores, i.e., soft stool, indicate normal to fast transit. BSC scores correlate well with outcomes of scintigraphy imaging or the radio-opaque marker method and to a lesser extent with defecation frequency [44].

### 2.6. Colonic Transit and Metabolic Health

The role of colonic transit in metabolic health is not well studied. A meta-analysis of 21 cross-sectional studies concluded that GI symptoms such as diarrhea, but not constipation, were associated with increased BMI [45]. Small scales studies assessing colonic transit are less conclusive. Studies reported slower [46,47] as well as faster colonic transit [39] in obese compared to lean individuals. In T2DM, constipation is one of the most common reported GI symptoms [48] and underlying processes are likely to be related to the higher occurrence of autonomic and enteric neuropathy in T2DM [49]. Furthermore, colonic transit may play an indirect role in metabolic health via its reciprocal relationship with the gut microbiota, which is discussed in following section.

### 2.7. Gut Microbiota and Colonic Transit

The colon is the ecological habitat of trillions of bacterial cells termed the gut microbiota. Species belonging to the phyla *Firmicutes* with around 200 Gram-positive genera (incl. *Clostridium*, *Eubacterium*, *Faecalibacterium*, *Lactobacillus*, *Roseburia* etc.) and *Bacteriodetes* with around 20 Gram-negative genera (incl. *Bacteriodes*, *Prevotella* etc.) are the most abundant (>80%). Less abundant species belong to the phyla *Actinobacteria* (i.e., *Bifidobacteria*), *Proteobacteria* (i.e., *Desulvibrio*, *Escherichia*) and *Verrucomicrobia* (i.e., *Akkermansia muciniphila*) [50]. The symbiotic relationship between the microbiota and the human host plays a role in many physiological functions within the energy metabolism, metabolic cross-organ signaling, gut barrier integrity and (mucosal) immune system [51]. Although a healthy gut microbiota composition remains yet to be defined, evidence from animal and human studies suggests that alterations of gut microbiota contribute to the etiology of obesity and T2DM [52,53]. Microbial metabolites such as short chain fatty acids (SCFA) interact with metabolic signaling and inflammatory pathways on intestinal and systemic level [4,54]. Hence, they physiologically link the gut microbiota composition and activity with the host metabolic phenotype and consequently to metabolic disorders. In T2DM, fecal microbiota profiles have been reported to be associated with a reduced abundance of *Bifidobacteria* [55,56], *Akkermansia muciniphila* [57] and butyrate-producing bacteria such as *Roseburia* and *Faecalibacterium prausnitzii* [58,59,60,61,62]. *F. prausnitzii* may be an indicator for gut health [63] and might be involved in low-grade inflammation in obesity and T2DM [64]. The obese microbiota is characterized by a low abundance of certain taxa i.e., *Akkermansia* [62,65] and a reduced overall microbial diversity [66]. With respect to chronic metabolic diseases, modulation of gut microbiota composition and activity might consequently be a potential target for prevention or treatment opportunities.

Colonic transit time is an important host factor shaping the microbiota ecosystem since it regulates water and nutrient availability as well as the rate of luminal washout [67,68]. First observations on the relation between microbiota composition and GI transit stem from germ-free (GF) rodent models, which showed a delayed GE rate and delayed whole gut transit compared to conventionally raised animals [69]. Re-colonization with *Lactobacillus* and *Bifidobacterium* could normalize GI transit in GF rodents [70,71,72]. The gut microbiota might affect gut motility via different mechanisms. Microbially-derived metabolites and molecules such as SCFA, lipopolysaccharide (LPS), secondary bile acids and methane may affect colonic motility via neural and humoral (i.e., GLP-1, PYY, motilin, serotonin) pathways [73,74]. Vice versa, colonic transit may influence gut microbiota composition and its functionality. Early human studies with loperamide-induced delayed or sienna-induced accelerated colonic transit showed that fecal microbial mass either increased with accelerated, or decreased with delayed colonic transit [75,76]. With more sophisticated 16S rRNA sequencing techniques available, several studies have revisited the interaction between colonic transit and the gut microbiota. In a population analysis with 1335 participants, stool consistency (measured by BSC score) was the top covariate explaining most of the microbiota community variation observed [3]. In healthy women, softer stool was associated with increased abundance of *Bacteroides* while firmer stool was associated with increased abundance of methane-producing archaea and increased species richness [77]. A prospective cohort study including 1126 participants reported no association between stool consistency and species richness but a similar positive correlation between abundance of methanogens (*Methanobrevibacter*), *Clostridiaceae* and firmer stool consistency. Interestingly, *F. prausnitzii* was strongly associated with softer stool consistency [78]. A recent cross-sectional study with 85 overweight to obese participants with increased risk for metabolic syndrome showed that slower colonic transit was associated with increased *Methanobrevibacter* and reduced *F. prausnitzii* abundance as well as an increased production of microbial protein catabolism end-products (i.e., p-cresol, indole) [79]. The authors conclude that with a delayed colonic transit, indigestible carbohydrates are more and more depleted driving bacteria to switch to protein fermentation. However, the authors did not investigate colonic transit and gut microbiota in relation to the metabolic phenotype of the participants.

In summary, a slow colonic transit is associated with a methanogenic profile and increased bacterial protein catabolism. Slow transit may be accompanied by carbohydrate deprivation, and a subsequent microbial energy metabolism shift towards protein fermentation. This may result in an increase of metabolites potentially detrimental for metabolic health (i.e., branched-chain fatty acids (BCFA), ammonia and aromatic derivatives of amino acids) [80]. On the other hand, faster transit was associated with higher *F. prausnitzii* abundance, which is associated with gut health and reduced low-grade inflammation. Thus, modulating colonic transit might be a potential target to counteract microbial dysbiosis related to chronic metabolic diseases.

## 3. Dietary Fibers

Dietary fibers are a heterogeneous group of food compounds. Generally, dietary fibers are indigestible carbohydrates (i.e., cellulose, hemicelluose, β-glucans, pectins, gums fructans and resistant starch) and lignins intact and intrinsic in fruits, vegetables, legumes and cereals. Chemically, dietary fibers include non-starch polysaccharides such as cellulose, hemicellulose, β-glucans, polyfructoses (i.e., inulin), natural gums and heteropolymers (i.e., pectin) as well as natural or synthetically produced oligosaccharides (i.e., fructo-oligosaccharides (FOS), galacto-oligosaccharides (GOS)) [81]. These compounds vastly differ in their structural, physical and chemical properties, namely water solubility, viscosity, binding and bulking ability and fermentability [82].

### 3.1. Effect of Insoluble Dietary Fibers on Glycemia

Insoluble fibers include cellulose, hemi-celluloses and lignin, which are major components of plant cell walls. Insoluble fibers are the main constituent of dietary fiber fractions in cereals, grains, vegetables and fruits. However, it is important to note that most fiber–rich foods contain soluble, insoluble, (non-) fermentable fibers in varying ratios [83]. Foods rich in insoluble fibers such as whole grains and cereals are consistently associated with a reduced risk of developing T2DM in observational studies [6,84,85,86,87]. A recent meta-analysis with more than 15,000 participants concluded that an increase in cereal fiber intake of 10 g/day decreased the risk of developing T2DM by 25% independent of BMI [7]. In line, a meta-analysis of 17 prospective cohort studies reported that an increase in cereal fiber intake of 2 g/day reduced the risk to develop T2DM by 6% (RR 0.94, 95% CI 0.93–0.96) [88]. Only a few intervention studies investigated the impact of foods rich in insoluble fiber on GI transit and glucometabolic effects. In an acute crossover study, 14 healthy women consumed either bread enriched with 10.4 g wheat fibers or 10.6 g oat fibers or a white bread as a control [89]. Intake of both high fiber breads increased early postprandial insulin peak and reduced postprandial glucose concentration, but stool consistency did not change. In another crossover study, 50 overweight or obese participants fulfilling one or more metabolic syndrome criteria consumed a whole grain diet (179 ± 50 g/day) or a control diet with refined grain for 8 weeks. Circulating low-grade inflammatory markers and body weight (from 85.4 kg ± 13.4 kg to 85.2 kg ± 13.4 kg, *p* < 0.001) were reduced after the whole grain diet, which correlated with a reduced total energy intake. The whole grain intervention had no effect on fasting blood glucose, insulin, lipid or GLP-1 concentrations and no major changes in fecal gut microbiota or whole gut transit measured by radio-opaque markers were observed [90].

#### Underlying Mechanism: A Role of GI Transit?

To date, it is not fully understood which mechanisms are responsible for the beneficial effects of insoluble fiber intake on health. With regards to GI transit, it is generally accepted that laxation is one of the major health benefit of insoluble and cereal fiber intake. Insoluble fibers increase fecal bulking and stool water content due to their high water-binding capacity which in turn mechanically stimulates mucus secretion and peristalsis [91,92,93]. A weighted regression analysis of 65 interventions studies in healthy participants showed that an increase of cereal and wheat fiber of 1 g/day increased stool wet weight by 3.9 g/day. Interestingly, only in individuals with a GI transit time above 48 h, the increased intake of 1 g/day cereal and wheat fiber led to a decrease in colonic transit of 0.78 h/day [91]. Fiber-induced fecal bulking affects the rheological structure of the food matrix which might impact the bioavailability and bio-accessibility of macronutrients within the matrix [94]. Thus, macronutrients might be less accessible for digestion and absorption and consequently reduce energy intake, which may contribute to the observed beneficial effects on metabolic health. Besides, the replacement of available carbohydrates by indigestible carbohydrates per se might reduce energy content of the diet. Further, microbial modulation might also occur due to fiber-induced changes of colonic habitat such as increased water availability and trapping of nutrients within the stool matrix. Microbial derived SCFA have been proposed as one of the potential beneficial mechanisms explaining the improved insulin sensitivity with diets high in insoluble fiber [95]. However, the degree of fermentability of insoluble fibers and the possible effect on metabolic health is controversial [96,97,98].

It should be kept in mind, that most sources of insoluble fiber are consumed as whole grains or partly processed cereal grains, which may differ in functionality (grain source and refining procedure). Furthermore, they also contain varying amounts of soluble fibers and bioactive phytochemicals, such as polyphenols, that might also contribute to the observed health benefits [99]. It is evident that further human studies investigating the relation between insoluble fiber intake, GI transit and metabolic health are warranted.

### 3.2. Effects of Soluble, Viscous Fiber and Postprandial Glycemia

Soluble, viscous fibers include polysaccharides such as plant-derived pectins, β-glucans, psyllium/ispaghula husks, natural gums, galactomannans and alginates. Once dissolved in water, viscous fibers form gels and/or thicken, a physico-chemical characteristic that may impact intestinal motility and absorption rates of glucose, triglycerides and cholesterol [100]. Randomized, controlled studies reported that viscous and/or gel-forming fibers may improve glycemic and insulinemic responses [101]. In fact, the European Food Safety Agency (EFSA) authorized the health claim that consumption of 4 g of β-glucans (derived from barley and oats) per 30 g of available carbohydrates is sufficient to reduce postprandial glucose concentration in the range of clinical significance [102]. The viscous fiber psyllium has been reported to lower postprandial glucose concentrations and improve insulin sensitivity in healthy, obese and T2DM individuals. This positive effect on postprandial glycemia was reported in intervention studies ranging from 6 weeks to 6 months with doses of 10–14 g psyllium per day [103]. A meta-analysis of 4 studies (duration 2 to 24 weeks) in T2DM individuals showed that psyllium intake decreased fasting glucose concentration by 37 mg/dL and reduced HbA1c (−10.6 mmol/mol) compared to placebo [104]. Similar, 6-week supplementation of 10 g/d natural partly hydrolyzed guar gum reduced fasting glucose and insulin concentration in healthy men [105] and HbA1c concentration in T2DM patients [106]. However, other studies with hydrolyzed guar gum supplementation in T2DM patients reported no effect on glycemia [107,108]. Acute reduction of subjective appetite rating and acute energy intake was reported after intake of pectins (mean dose 14.2 g/day and 4.8 g/day, respectively) and β-glucans (mean dose 6.2 g/day and 5.8 g/day, respectively) compared to the control food [109].

#### Underlying Mechanisms: Importance of Viscosity

It has been suggested that viscosity is crucial to exert beneficial fiber-specific effects on glucose homeostasis and appetite regulation. High food viscosity induces gastric distension i.e., feeling of fullness [110], delays GE and/or physically prevents absorption of nutrients in the small intestine [111,112]. Pharmacologically or fiber-induced delayed GE rate was associated with reduced feelings of hunger and increased satiety [113]. Hence, this suggests that viscous fibers might improve glycemic control by delaying GE rate. Table 1 gives a summary of available human intervention studies on viscous fiber intake on GE rate and postprandial glycemia. Early scintigraphy studies showed that both healthy volunteers and T2DM individuals had a delayed GE after the consumption of 20 g apple pectins/day for 4 weeks, which was accompanied by reduced postprandial glucose concentrations only in T2DM individuals [114,115]. In healthy volunteers, acute intake of liquid drink supplemented with 2.5 g pectin delayed GE measured by ^13^C-acetate breath test, but no changes were observed in postprandial glucose concentrations [116]. Acute intake of 5 g sodium-alginate with a liquid meal reduced postprandial glucose and insulin concentrations in T2DM patients, which was correlated with a delayed GE assessed by scintigraphy [117]. In another acute study, intake of high viscous β-glucans attenuated the early postprandial glucose response and delayed GE rate in healthy volunteers compared to the less viscous ß-glucan [118]. In contrast, acute supplementation of 1.7 g psyllium to a solid meal neither reduced GE, nor affected postprandial glucose and insulin concentrations in healthy volunteers [119]. Acute intake of β-glucans with low or high viscosity in an oat bran beverage resulted in higher postprandial GLP-1 and PYY concentrations and faster GE rate with the low-viscosity compared to high viscosity beverage in healthy volunteers [120]. In contrast, acute meal supplementation of 23 g psyllium led to reduced postprandial glucose and GLP-1 concentrations in healthy volunteers as compared to an isocaloric low fiber meal. The authors conclude that a psyllium-dependent increase in luminal viscosity physically impairs the efficient stimulation of L-cells and concomitant GLP-1 and PYY release [121].

Post-gastric events might also contribute to the observed effects of viscous fiber on postprandial glycemic control, GE and satiety. There is limited evidence that viscosity is maintained throughout the small intestine, since the luminal content is diluted with gastric secretions during passage from stomach to duodenum. Besides, the degree of viscosity depends on structural and chemical composition of the fiber type and can differ across pH gradients in the GI tract [122]. Viscous, gel-like chyme can, to some extent, bind and thereby limit diffusion of nutrients within the gel matrix. This reduces the contact of nutrients with digestive enzymes and absorption from the intestinal epithelium [100,112]. In rat intestinal cells, incubation with β-glucans suppressed glucose uptake, which was accompanied by reduced expression of SGLT1 and GLUT2 [123]. Also, postprandial intestinal contractility is prolonged (indicating a delayed small intestine transit) after 5 g guar gum supplementation to solid or liquid meals in healthy individuals [124]. On the other hand, several acute studies reported no differences in small intestinal transit time after ingestion of guar gum or psyllium as measured by indirect hydrogen breath tests [119,125,126].

Hence, there is a robust link between acute viscous fiber intake and delayed GE, however this is not consistently associated with an improved postprandial glucose response. Differences in methodology for measuring GE rate, interactions with other meal compounds, and degree of viscosity might play a role in the variability of the interaction between viscous fiber, GE and glycemia. Evidence from animal and in vitro studies suggests that viscous fiber might interfere with intestinal glucose uptake and absorption. This might partly explain the observed beneficial effects on glycemia in humans. However, well-controlled human studies are warranted to further elucidate the underlying mechanism of viscous fiber on GE rate, postprandial glycemia and intestinal glucose absorption.

### 3.3. Effects of Soluble, Non-Viscous Fibers on Glycemia

Soluble, non-viscous fibers are fructans (inulin, FOS, GOS, xylo- and arabinoxylan oligosaccharides (A(XOS)), resistant starches and analogous polysaccharides such as polydextrose that are fermentable by the colonic gut microbiota [127]. Some soluble fibers such as inulin, FOS, GOS and XOS are classified as indigestible prebiotics defined as ‘a substrate that is selectively utilized by host microorganisms conferring a health benefit’ [128]. Intake of inulin, FOS and GOS results in an increased abundance of bacterial species associated with beneficial health effects such as *Bifidobacteria* and *Lactobacillus* [129,130,131,132]. These genera have been associated with various beneficial effects including strengthening of gut barrier function, improving host mucosal immunity, increased SCFA production and protection against opportunistic gut pathogens [132]. Fructans (inulin, FOS) and GOS have been extensively studied in diet-induced obese or ob/ob rodent models. The supplementation with GOS and FOS in these rodent models improved glucose homeostasis, reduced serum lipids and a reduced weight gain during a high-fat diet [82,133,134]. With respect to glycaemia, human intervention trials are less conclusive and either report improved postprandial glucose and insulin concentrations in healthy and obese volunteers [135,136,137] or no changes [138,139]. A recent systematic review summarized 20 randomized controlled trials with 607 healthy, obese and T2DM patients with inulin-type fructan ITF (FOS, GOS, inulin and mixes) supplementation ranging from 7.4 g to 30 g/d during a period of 20 days up to 6 months. Overall analysis showed that fasting insulin and glucose concentrations were only reduced in T2DM individuals [140].

#### Underlining Mechanisms: A Role for Microbial Functionality?

The potential underlying mechanism of prebiotics may involve the modulation of microbial metabolite production (SCFA, secondary bile acids) and reduction of bacterial constituents (LPS). SCFA and bile acids are tightly involved in energy homeostasis, insulin signaling, fat accumulation and inflammatory signaling, as review elsewhere [4,141,142,143,144]. Besides, these microbial metabolites might also indirectly affect GI transit as described in Section 2.7. Animal and in vitro studies reported that many bacterial metabolites i.e., SCFAs, hydrogen, LPS and secondary bile acids interact with enteric nerves and smooth muscles function possibly stimulating GI transit time [73]. The few existing human studies indicate that inulin might potentially improve colonic transit and potential delay GE rate. Inulin intake promotes bowel movements and softer stool consistency indicating a faster colonic transit, however not in the same magnitude as insoluble dietary fiber [145]. In a crossover studies with healthy men, daily inulin-enriched (11% inulin) pasta intake over 5 weeks decreased GE rate and decreased fasting glucose concentrations [146]. Yet, there is no data on other prebiotics available, making it difficult to draw valid conclusions. To sum up, well-controlled human intervention studies are warranted to substantiate the effect of prebiotic fibers on improvement of postprandial glycemia and the potential involvement of GI transit.

## 4. Conclusions

**Figure 1:**
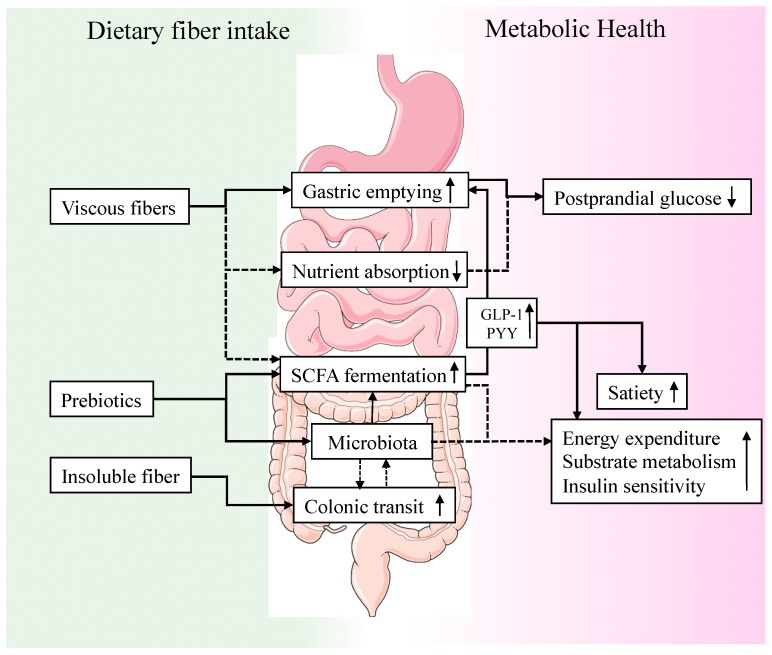
The complex relationship between dietary fiber intake, the gastrointestinal tract and host metabolism. Viscous fibers increase gastric emptying rate, may inhibit nutrient absorption and contribute to SCFA fermentation. These effects may lead to a reduced postprandial glucose appearance and an increased release of incretin and satiety-stimulating hormones (GLP-1, PYY), which might influence energy intake and peripheral tissue metabolism. Prebiotics modulate microbiota composition and SCFA production thereby affecting energy homeostasis and insulin sensitivity. Insoluble fibers are most effective in increasing colonic transit time thereby possibly affecting microbiota composition, and vice versa microbial metabolites may stimulate colonic motility. Solid lines indicate well-studied effects of dietary fiber, dashed line indicate more controversial findings. Abbreviations: SCFA short-chain fatty acids; GLP-1 glucagon-like peptide 1; PYY peptide YY.

To summarize and conclude, there are site-specific effects of GI transit on postprandial glucose homeostasis and metabolic health (Figure 1). GE and small intestinal transit are mainly involved in central appetite signaling, initial glucose appearance in the circulation and gut peptide secretion. The underlying mechanisms of insoluble fibers in improving glucose homeostasis and reducing T2DM risk may (partly) be related to effects on GI transit. Modulation of GI transit, which in turn may also affect the microbiota composition, might be an underlying mechanism and should be considered in future human intervention studies. Viscous fibers can delay postprandial glycemia mediated by changes in gastric emptying, yet the role of gut peptide secretion in this process is not fully understood. Further studies are warranted to understand the underlying mechanisms linking gut motility, dietary fiber and glucose homeostasis.

## Figures and Tables

**Table 1:** Summary of human intervention studies with viscous dietary fiber on GE rate and postprandial glucose metabolism.

Participants	Design	Intervention	Method	GE Rate	Metabolic Outcomes	Reference
13 healthy adults (6 men, 7 women)	2-week isocaloric low-fiber diet followed by 4-week low fiber diet + supplement	20 g/day apple pectin baked in muffins or 20 g/day cellulose supplement as control fiber	Scintigraphy Solid meal (545 kcal, 74% CHO, 23% Protein, 1% fat)	↑ T_1/2_	↔ glucose	[115]
12 non-insulin dependent T2DM patients (7 men, 5 women)	2-week isocaloric low-fiber diet followed by 4-week low fiber diet + supplement	20 g/day apple pectin baked in muffins	Scintigraphy Solid meal (690 kcal, 43% CHO, 43% Protein, 23% fat)	↑ T_1/2_	↓ iAUC glucose	[114]
7 male T2DM patients (BMI 20–30 kg/m^2^)	Acute crossover study	5 g sodium-alginate, control drink without supplement	Scintigraphy Semi-solid meal (340 kcal, 48% CHO, 13% protein, 39% fat)	↑ T_1/2_	↓ postprandial peak insulin ↓ postprandial peak glucose	[117]
10 healthy men	Acute crossover study	2 g agar or 4 g pectin, control drink without supplement	^13^C-acetate breath test Semi-solid meal (400 kcal, 32% CHO, 8% protein, 39% fat)	↑ T_1/2_ ↑ T_lag_	↔ AUC glucose	[116]
10 healthy adults (4 men, 6 woman)	8 Acute crossover study	Pasta meal supplemented with 1.7 g psyllium and with or without added sunflower oil	Paracetamol absorption High-fat solid meal (510 kcal, 45% CHO, 1% protein, 52% fat) Low-fat solid meal (240 kcal, 96% CHO, 3% protein)	↔ AUC paracetamol	↔ glucose ↔ insulin ↔ GLP-1	[119]
15 healthy adults (3 men, 12 women)	Acute crossover study	High molecular weight 12.8 g, β-glucan (25% purity), low molecular weight 3.6 g β-glucan (75% purity), control without supplement	^13^C-acetate breath test Liquid meal (189–192 kcal, 60–67% CHO, 7–10% protein, 27–29% fat)	↑ T_1/2_ ↑ T_lag_	↓ iAUC_0–60min_	[118]

CHO carbohydrate; T_1/2_ gastric emptying half time; T_lag_ initial gastric emptying rate; AUC area under the curve; iAUC incremental area under the curve; % Percentage of total energy intake.
